# Detection and Potential Utility of C-Reactive Protein in Saliva of Neonates

**DOI:** 10.3389/fped.2014.00131

**Published:** 2014-11-21

**Authors:** Anjali Iyengar, Jessica K. Paulus, Daniel J. Gerlanc, Jill L. Maron

**Affiliations:** ^1^Division of Newborn Medicine, Floating Hospital for Children at Tufts Medical Center, Boston, MA, USA; ^2^Tufts Medical Center, The Institute for Clinical Research and Health Policy Studies, Boston, MA, USA; ^3^Tufts Clinical and Translational Science Institute, Tufts University, Boston, MA, USA; ^4^Enplus Advisors Inc., Cambridge, MA, USA; ^5^Mother Infant Research Institute (MIRI) at Tufts Medical Center, Boston, MA, USA

**Keywords:** biomarker, C-reactive protein, inflammation, neonates, saliva

## Abstract

**Objective:** We aimed to detect C-reactive protein (CRP) in neonatal saliva and evaluate its diagnostic utility.

**Study Design:** Salivary and serum samples (*n* = 89) were collected from 40 neonates. Salivary CRP levels were determined using an enzyme-linked immunosorbent assay; serum CRP was measured per hospital protocol. Correlation coefficients with 95% confidence intervals and robust linear regression measured association while receiver–operator characteristic curves described the accuracy of salivary CRP in discriminating abnormal serum CRP thresholds of ≥10 and 5 mg/L. Corresponding sensitivities and specificities were calculated for these salivary cutpoints.

**Results:** The area under the curve for salivary CRP in predicting serum CRP levels of ≥10 and 5 mg/L were 0.81 and 0.76, respectively. The corresponding sensitivity and specificity for raw salivary CRP to discriminate a serum CRP of ≥5 mg/L was 0.54 and 0.95, respectively. The corresponding sensitivity and specificity for raw salivary CRP to discriminate a serum CRP of ≥10 mg/L was 0.64 and 0.94, respectively. A statistically significant correlation was observed between serum and salivary CRP (*r* = 0.62, *p* < 0.001).

**Conclusion:** C-reactive protein is detectable in neonatal saliva and can predict abnormal serum CRP thresholds. Salivary CRP analysis represents a feasible screening tool for detecting abnormal serum CRP levels.

## Introduction

C-reactive protein (CRP) is an extensively utilized biomarker for monitoring sepsis, post-surgical complications, and inflammation in the pediatric and neonatal populations (1). Produced in the liver in response to IL-6, CRP levels rise rapidly during infection and/or inflammation (1, 2). However, a current limitation to the usefulness of serial CRP monitoring in the neonatal population is its reliance on frequent blood draws in a vulnerable population with limited blood volumes. Developing a non-invasive assay for the quantification of CRP in neonates could reduce neonatal side-effects and ultimately improve its clinical utility.

Saliva is an excellent biofluid to non-invasively monitor the neonate. Filtered from whole blood in the salivary glands, saliva is an important reservoir of systemic proteins and immunoglobulins, electrolytes, nucleic acids, microorganisms, toxins, and drugs (3). The collection of neonatal saliva for protein analysis is well documented. Studies examining salivary cortisol levels in the sick and preterm infant are plentiful, as are studies examining testosterone, amylase, and various antibody/antigen responses and their association with systemic disease (4–9). Recent animal data have demonstrated the correlation of serum and salivary CRP measurements in both diseased and healthy animals (10). Additionally, there is emerging literature supporting the use of saliva as a surrogate biofluid for serum CRP monitoring in healthy adults (11, 12). However, the clinical utility of salivary CRP monitoring in the newborn is currently unknown. The objective of this study was to determine if salivary CRP was detectable in neonatal saliva and to examine its diagnostic capabilities and limitations in the clinical setting.

## Materials and Methods

### Study population

This was a prospective Tufts Medical Center (TMC) Institutional Review Board (IRB) approved study. Parents of infants hospitalized in the TMC Neonatal Intensive Care Unit (NICU) from August 2011 to October 2012 who required serial CRP levels as part of their routine care were asked to participate. There were no exclusion criteria based on gender, genetic disorder, disease process, or clinical status.

### Procedures

Neonatal salivary samples were collected within 4–12 h of clinically indicated serum CRP levels. For those infants receiving enteral nutrition, every attempt was made to collect saliva 1 h prior to a feed to avoid breast milk or formula contamination. Salivary samples were collected with protocols previously established in our laboratory (13). Samples were obtained over 5–15 s using a 1 mL syringe, wings removed, attached to low-wall suction. The syringe was weighed pre- and post-salivary collection to determine salivary volume. Saliva was then placed in 65 μL of a protease inhibitor (SigmaFAST™, St. Louis, MO, USA) and RNAprotect Saliva (Qiagen™, Venlo, Limburg, Netherlands) in a 1:10 concentration for salivary protein stabilization (13–15). Samples were vortexed, placed on ice, and subsequently stored at −80°C for several weeks up to 8 months before processing.

Total protein concentration in each sample was determined with the NanoDrop™ Spectrophotometer 1000 (Thermo Fisher Scientific, Inc., Waltham, MA, USA). Salivary CRP concentrations were determined with a commercial electrochemiluminescence immunoassay (MesoScale Discovery™, Rockville, MD, USA) per manufacturer’s instructions. All samples were run in triplicate and plotted against a standard curve for CRP quantification. Serum CRP measurements were determined in the TMC hospital laboratory using the UniCel DxC 600i immunoassay analyzer (Beckman Coulter, Inc., Brea, CA, USA).

### Statistical analysis

Statistical analyses used the SAS statistical package, version 9.2 (SAS Institute, Cary, NC, USA) and R (version 2.15.1). Medians and interquartile ranges were used to summarize demographic and clinical characteristics of the patients. Spearman correlation coefficients and their 95% confidence intervals (CI) were used to measure the association between salivary protein and volume, serum CRP and raw salivary CRP, and serum CRP and salivary CRP normalized with either total protein concentration or volume. All associations fit a robust linear regression model. We minimized the minimum absolute deviations of the residuals (MAD) using the method of iterative least squares (16). This was calculated using the “lm” function in the R Project for Statistical Computing (17).

The discrimination of the salivary CRP levels for predicting abnormal serum CRP levels were calculated and compared using the area under the ROC curve (AUC) and corresponding CI using the method of Delong et al. (18). To examine the practical application of the salivary CRP levels, the sensitivity and specificity, and corresponding 95% CI, using 2000 bootstrap replications, were calculated for cut-offs based on the upper limit of each range. The sensitivities, specificities, and corresponding receiver–operator characteristic (ROC) curves were created using the “pROC” package in R. Optimal salivary CRP cutpoints were determined using Youden’s J-statistic (19). To address the fact that multiple samples were collected from 22 out of 35 of the patients in the study population, an additional sensitivity analysis was conducted on the dataset by randomly selecting one paired measurement from these patients.

## Results

There were 40 infants initially enrolled in this study. As designated by the study design, five patients were excluded from the analysis because their salivary samples were obtained >12 h from the associated serum CRP level. Aside from this, no other patients or samples were excluded for any reason. From the remaining 35 patients, 89 salivary samples were ultimately included in the final analysis. Subjects’ gestational ages and birth weights ranged from 23 to 42 weeks and 490 to 3950 g, respectively. The majority of serum CRP levels were obtained for post-operative monitoring (44/89 salivary samples), followed by necrotizing enterocolitis (NEC) or spontaneous intestinal perforation (SIP) (33/89 salivary samples), and infectious disease (12/89 salivary samples) (Table 1).

**Table 1:** **Demographic characteristics of study population**.

Total patients (*n*)	35
Birth weight (g)	1774 (946–2253)^a^
Current weight (g)	2102 (1375–2625)^a^
Gestational age (weeks)	30.4 (26–34.8)^a^
PCA at evaluation (weeks)	34.5 (31.6–38.1)^a^
Male:Female	1.8:1
Total salivary samples (*n*)	89
Salivary samples per patient	2 (1–3)^a^
Serum CRP (mg/L)	106.1(4.8–127.2)^a^
Raw Salivary CRP (ng/mL)	3.1 (0.4–22)^a^
Salivary protein concentration (mg/dL)	3.1 (2–5.9)^a^
Salivary volume (μL)	35.4 (18.2–64.9)^a^

*^a^Median (25th–75th interquartile range)*.

**Figure 1:**
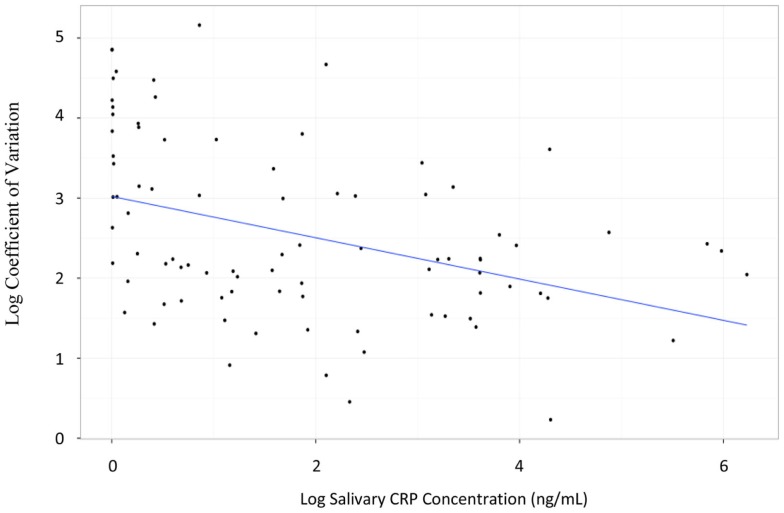
**Graph illustrating the logarithmic relationship between coefficient of variation and salivary CRP concentration**. The transformation used was log(*x*) − 1.

Salivary CRP was detected in 97% of the samples analyzed (86/89) with a median sample volume of 35.4 μL (IQ range = 18.2–64.9 μL). In contrast, the minimal volume needed for the measurement of serum CRP was 200 μL. The median salivary CRP concentration was 3.1 ng/mL (IQ range = 0.4–22 ng/mL); the median serum CRP concentration was 106.1 mg/L (IQ range = 4.8–127.2 mg/L). The lower limit of detection (LLOD) for salivary CRP was 4.5 pg/mL. Intra-assay coefficients of variation (CVs) at low (10 pg/mL) and high (394 474 pg/mL) concentrations were 56 and 9%, respectively. Inter-assay CVs at low (9 pg/mL) and high (752 124 pg/mL) concentrations were both 10%. The CV of all triplicate samples was dependent upon CRP concentration. Samples with CRP concentrations at the lower limit of our assay detection had much higher CVs than samples within range (CVs ranged from 1.9 to 86.7%; Figure 1).

**Figure 2:**
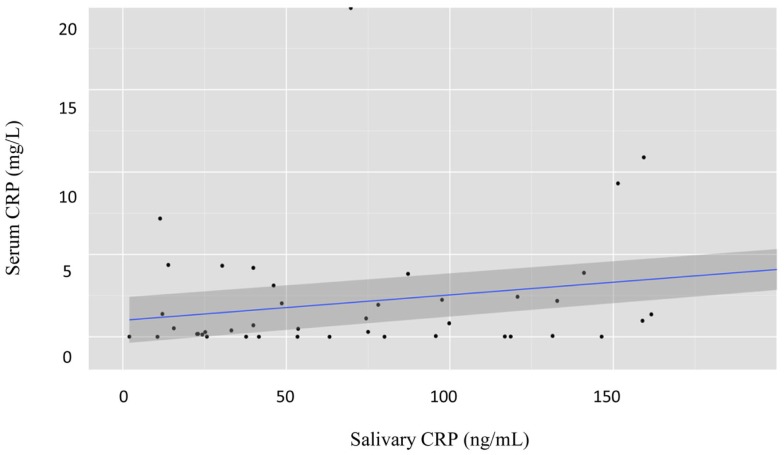
**Graph illustrating the estimated parameters from a regression of salivary CRP levels on serum CRP levels**. Both salivary CRP and serum CRP were power transformed; salivary CRP was transformed by taking the natural log of salivary CRP (ng/mL) + 1. Serum CRP was transformed using a Box–Cox transformation with a lambda of 0.12; *n* = 89 salivary samples.

There was a statistically significant correlation between serum and raw salivary CRP concentrations [*r* = 0.62, *p* < 0.001 (95% CI: 0.47–0.73); Figure 2]. However, given varying volumes of saliva obtained with each sample, we normalized the data. Historically, salivary volume has been used to normalize analyte concentration across samples (20–25). However, in our study, salivary protein concentration was weakly associated with sample volume [*r* = 0.35, *p* = 0.01 (95% CI: 0.11–0.55)]. Thus, we examined the relationship of raw salivary CRP levels to protein concentration and volume separately. Raw salivary CRP concentrations were more strongly associated with protein concentration as compared to volume [*r* = 0.62, *p* < 0.001 (95% CI: 0.48–0.74) and *r* = 0.52, *p* < 0.001 (95% CI: 0.33–0.69)]. Based on these data, we normalized salivary samples using total protein concentration. A statistically significant association was also observed between serum CRP and the protein-adjusted salivary CRP [*r* = 0.57, *p* < 0.001 (95% CI: 0.40–0.69)]. Similar associations were seen between serum CRP and raw and protein-adjusted salivary CRP in a sensitivity analysis limited to one randomly selected sample per patient [*r* = 0.68, *p* < 0.001 (95% CI: 0.44–0.82) and *r* = 0.60, *p* < 0.001 (95% CI: 0.33–0.78), respectively].

**Figure 3:**
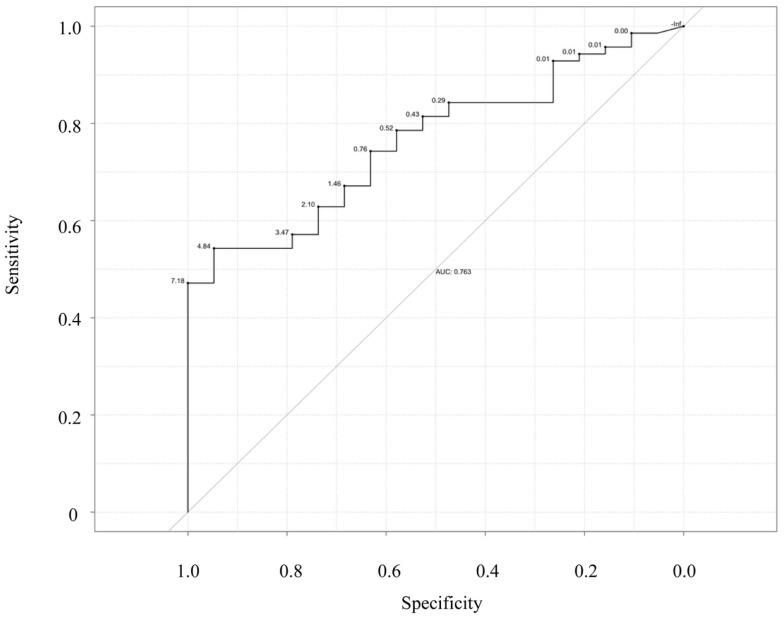
**Receiver–operator characteristic (ROC) curve of raw salivary CRP for predicting serum CRP ≥5 mg/L**. Sensitivity is plotted against specificity for salivary CRP thresholds between 0 and 7.18 ng/mL; AUC = 0.76.

**Figure 4:**
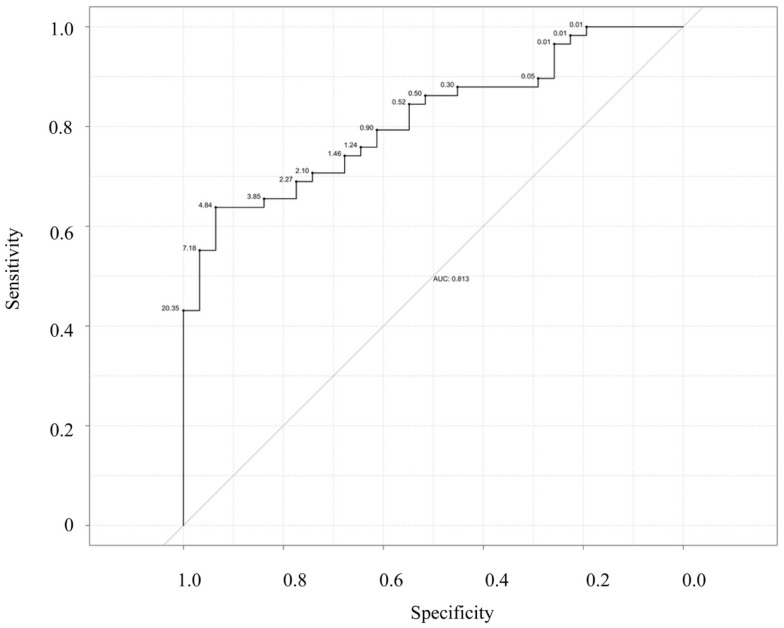
**Receiver–operator characteristic (ROC) curve of raw salivary CRP for predicting serum CRP ≥10 mg/L**. Sensitivity is plotted against specificity for salivary CRP thresholds between 0.01 and 20.35 ng/mL; AUC = 0.81.

Next, we examined the ability of salivary CRP to predict serum CRP thresholds of ≥5 and 10 mg/L (Figures 3 and 4). Raw salivary CRP demonstrated good diagnostic accuracy at predicting a serum CRP of ≥10 mg/L (AUC = 0.81, 95% CI: 0.72–0.90) and demonstrated fair diagnostic accuracy at predicting a serum CRP of ≥5 mg/L (AUC = 0.76, 95% CI: 0.66–0.87). Protein-adjusted salivary CRP demonstrated fair diagnostic accuracy at predicting a serum CRP of ≥10 and ≥5 mg/L [AUC = 0.78 (95% CI: 0.68–0.87) and 0.75 (95% CI: 0.65–0.86)], respectively. Again, similar AUC results for raw and protein-adjusted salivary CRP were seen in a sensitivity analysis limited to one randomly selected sample per patient. Raw salivary CRP demonstrated good and fair diagnostic accuracy at predicting a serum CRP of ≥10 and 5 mg/L [AUC = 0.86 (95% CI: 0.74–0.99) and 0.78 (95% CI: 0.63–0.93)]. Protein-adjusted salivary CRP demonstrated good and fair diagnostic accuracy at predicting a serum CRP of ≥10 and ≥5 mg/L [AUC = 0.82 (95% CI: 0.67–0.98) and 0.77 (95% CI: 0.61–0.92)].

Finally, statistically determined salivary cut-offs were generated optimizing sensitivity and specificity for raw and protein-adjusted salivary CRP. A raw salivary CRP concentration of 4.84 ng/L had a corresponding sensitivity and specificity of 0.64 and 0.94 in accurately predicting a serum CRP level of ≥5 mg/L and a corresponding sensitivity and specificity of 0.54 and 0.95 in accurately predicting a serum CRP of ≥10 mg/L (Table 2). A protein-adjusted salivary CRP concentration of 133.38 ng/mL had a corresponding sensitivity of 0.61 and specificity of 0.84 in accurately predicting a salivary CRP of ≥5 mg/L. A protein-adjusted salivary CRP cut-off of 166.11 ng/mL had a similar sensitivity and specificity (0.67 and 0.84, respectively) in predicting a salivary CRP of ≥10 mg/L.

**Table 2:** **Optimal salivary CRP cutpoints associated with maximal sensitivity and specificity in predicting abnormal serum CRP levels**.

Metric	Optimal salivary CRP cutpoint (ng/mL)^a^	Sensitivity (95% CI)^b^	Specificity (95% CI)^b^	AUC (95% CI)^c^
Raw salivary CRP predicting serum CRP ≥5 mg/L	4.84	0.54 (0.43–0.66)	0.95 (0.84–1.00)	0.76 (0.66–0.87)
Raw salivary CRP predicting serum CRP ≥10 mg/L	4.84	0.64 (0.52–0.76)	0.94 (0.84–1.00)	0.81 (0.72–0.90)
Protein-adjusted salivary CRP predicting serum CRP ≥5 mg/L	133.38	0.61 (0.50–0.73)	0.84 (0.68–1.00)	0.75 (0.65–0.86)
Protein-adjusted salivary CRP predicting serum CRP ≥10 mg/L	166.11	0.67 (0.71–0.97)	0.84 (0.55–0.79)	0.78 (0.68–0.87)

*^a^Optimal cutpoints were determined using Youden’s J-statistic (19)*.

*^b^Sensitivity and specificity confidence intervals were determined using 2000 bootstrap replications*.

*^c^Confidence intervals calculated using the method of Delong et al. (18)*.

## Discussion

To our knowledge, this is the first study to detect, quantify, and demonstrate that salivary CRP is a good measure of discrimination for clinically relevant serum CRP thresholds. Additionally, we observed the modest yet statistically significant association between CRP measured in neonatal saliva and serum, which has been supported in previously published research in healthy adults and animals (10–12). These data together illustrate the potential of salivary CRP as a routine screening assay for the at-risk newborn.

There is a strong need for better surveillance and monitoring for infants at risk of infection and post-surgical complications. Neonatal sepsis is estimated to occur in 1–21 newborns per 1,000 live births, resulting in significant morbidity and mortality. The risk of periventricular leukomalacia (PVL), a form of white matter brain injury associated with poor neurological and developmental outcomes, increases twofold for every case of neonatal bacterial sepsis (26, 27). Furthermore, estimated sepsis-associated mortality rates are as high as 69% in the premature neonatal population (26). CRP has been previously validated as a non-specific, but nevertheless, informative biomarker for the identification of newborns at risk for these sequelae. Current neonatal care guidelines for sepsis monitoring include frequent invasive surveillance methods and the use of prophylactic antibiotics. Non-invasive salivary diagnostics using a dichotomous variable salivary CRP screening test, could markedly improve our ability to care for these infants by alerting the care-giver to abnormal serum levels without the need for repeated phlebotomy.

Historically, the majority of published salivary reports have used volume and flow rate to normalize salivary analytes across samples (21–23). However, hydration status directly impacts salivary flow, and the use of volume for normalization may not be ideal, particularly in the neonatal population where collection of a standardized volume of saliva may not always be possible (24, 25). We addressed this issue by examining the relationship between salivary CRP levels with total protein concentration and found that raw salivary CRP levels showed the strongest predictive value of an abnormal serum CRP level. While there are obvious advantages to using the raw salivary CRP levels, including a more rapid interpretation of salivary CRP levels, future studies will need to be performed to assess the most accurate and clinically informative normalization method in the newborn.

Our feasibility study provides initial support for the quantification of salivary CRP in neonates using broadly available laboratory-based technology. A limitation of this pilot study is the range of inter- and intra-patient variation. We believe that these findings represent the technical limitations of the assay, specifically at the level of threshold detection. Variation between replicates sharply decreased at higher salivary CRP concentrations compared to samples with lower levels of the biomarker. In developing this non-invasive assay for infants with weights as low 490 g from as little as 2 μL of saliva, it is not unexpected that some of our samples had suboptimal performance. Furthermore, some variability can be attributed to the possible circadian variation observed between serum and salivary CRP, micro-injury to the tissue, localized inflammation of the mucosa, gestational age of the patient, mode of delivery, and salivary gland maturation (12, 27–30). However, emerging analytic devices, such as microfluidic immunosensor chips and lab-on-a-chip devices, have five times the sensitivity of traditional enzyme-linked immunosorbent assays (ELISAs) used in this study, and may be used in multiplex formats to detect dozens of biomarkers simultaneously from a single sample source (31–34). As these technologies continue to evolve, they have the potential to become incorporated into the NICU for rapid non-invasive assessment of the newborn. We believe that the variation seen between sample replicates at the level of detection in our study will be minimized with such platforms and that this current limitation will ultimately not pose as a barrier to integration into clinical care.

In conclusion, we have shown that salivary CRP is readily detectable in the neonate and demonstrates good accuracy at discriminating between clinically relevant serum CRP thresholds. Serial salivary CRP screening of the at-risk patient may represent a feasible and safe alternative to frequent serum sampling by alerting the care-giver to a potential infection, inflammatory process, and/or surgical complication in the neonatal population. Future studies establishing normative values across gestational age and weight, as well as incorporating related protein markers of sepsis and inflammation, may provide even more specific assessment of the at-risk newborn. Ultimately, integrating salivary biomarkers into neonatal care could provide the most effective and minimally invasive approach to accurate sepsis and inflammation screening in this vulnerable population.

## Conflict of Interest Statement

The authors have no financial disclosures. This study was supported only by Tufts Medical Center institutional funding. No honorariums were given.

## Abbreviations

AUC, area under the curve; CI, confidence intervals; CRP, C-reactive protein; CV, coefficient of variation; ELISA, enzyme-linked immunosorbent assay; IRB, Institutional Review Board; LLOD, lower limit of detection; NEC, necrotizing enterocolitis; NICU, Neonatal Intensive Care Unit; PVL, periventricular leukomalacia; ROC, receiver–operator characteristic; SIP, spontaneous intestinal perforation; TMC, Tufts Medical Center.
